# Temporal Brain MRI Changes From Extrapontine Myelinolysis to Central Pontine Myelinolysis: A Case Report

**DOI:** 10.7759/cureus.19318

**Published:** 2021-11-06

**Authors:** ChengYang Lee, ChingChung Ko

**Affiliations:** 1 Medical Imaging, Chi Mei Medical Center, Tainan City, TWN

**Keywords:** extrapontine myelinolysis, central pontine myelinolysis, neuroimaging, diffusion-weighted images, osmotic demyelination

## Abstract

We present a case that was initially misdiagnosed as drug-related extrapyramidal side effect. However, after the suspected causative drugs were withdrawn, the patient’s condition did not improve. Neuroimaging revealed osmotic demyelination syndrome with a temporal pattern of change from extrapontine myelinolysis to central pontine myelinolysis. Given the limited literature on this rare condition, further research to support clinical diagnosis and treatment is needed.

## Introduction

Osmotic demyelination syndrome is a common complication when treating electrolyte imbalance, especially serum sodium homeostasis [1]. Several groups are particularly susceptible to this complication, including patients with chronic alcoholism [1,2], patients with post-transplant status [3], women with pregnancy-related hyperemesis, and elderly or debilitated people. When hyponatremia is rapidly corrected with hypertonic saline, glial cells may not respond to osmotic changes in a timely manner. This can result in rapid movement of intracellular fluid into the extracellular space and lead to cell shrinkage and cell death. Oligodendrocytes have been shown to play a key role in the development of osmotic demyelination syndrome [4].

Osmotic demyelination syndrome is divided into two categories depending on the affected area: central pontine myelinolysis (CPM) and extrapontine myelinolysis (EPM). CPM and EPM can exist separately or coexist. Central pontine type lesions are usually located in the pons and the important affected structures include the corticospinal, corticobulbar, and corticopontine tracts at the basis pontis. During the early course of the disease, classical symptoms like dysarthria, dysphagia, flaccid quadriparesis, and spasticity are present when the respective anatomic structures are involved [1,5]. During the later course of the disease, the pontine tegmentum is also involved. Locked-in syndrome is a characteristic presentation of CPM, which refers to the situation where cognitive function and oculomotor function are preserved, but patients cannot perform other voluntary movements (e.g., movement of limbs, respiratory muscles). In extrapontine type osmotic demyelination syndrome, the basal ganglion is the important involved structure, including the putamen, caudate nucleus, thalamus, etc. EPM typically manifests as a Parkinsonian movement disorder, such as limb rigidity, dystonia, bradykinesia, or facial masking. As a result, it can be difficult to diagnose EPM from parkinsonism or drug-related extrapyramidal side effects [1,6].

## Case presentation

We present a case of a 44-year-old male patient who was taken to the emergency department for unrelieved upper abdominal pain for the previous two days. Other reported symptoms included hiccups, constipation, nausea, and a poor appetite. He denied any seizure episodes, change in consciousness, or fever. According to his past medical history, he underwent endoscopic endonasal transsphenoidal surgery for a sellar Rathke cleft cyst two years previously. In the emergency department, his vital signs were temperature of 35.6 ℃, heart rate of 85 bpm, blood pressure of 103/85 mmHg, and respiratory rate of 15 bpm. Biochemical testing revealed severe hyponatremia (100 mmol/L). Physical examination of the abdomen revealed no obvious abnormality. Under the assumption of a diagnosis of syndrome of inappropriate antidiuretic hormone secretion (SIADH) related hyponatremia, the patient was admitted to the general ward for further care.

**Figure 1:**
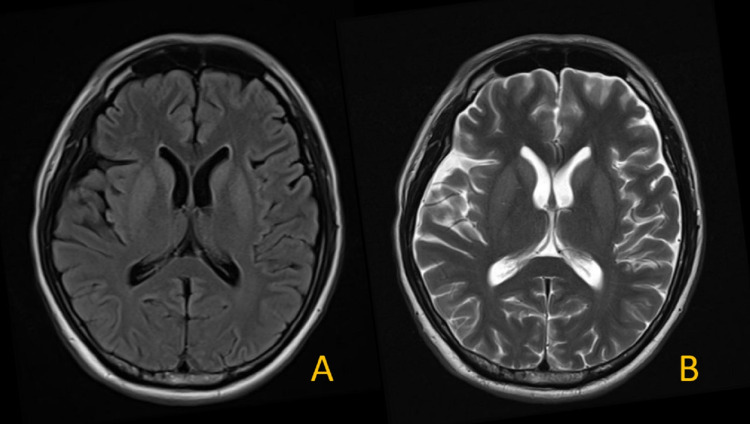
Brain MRI on day two of hospital admission. The axial FLAIR (A) and T2WI (B) images show no significant abnormalities. FLAIR: Fluid-attenuated inversion recovery; T2WI: T2 weighted image

**Figure 2:**
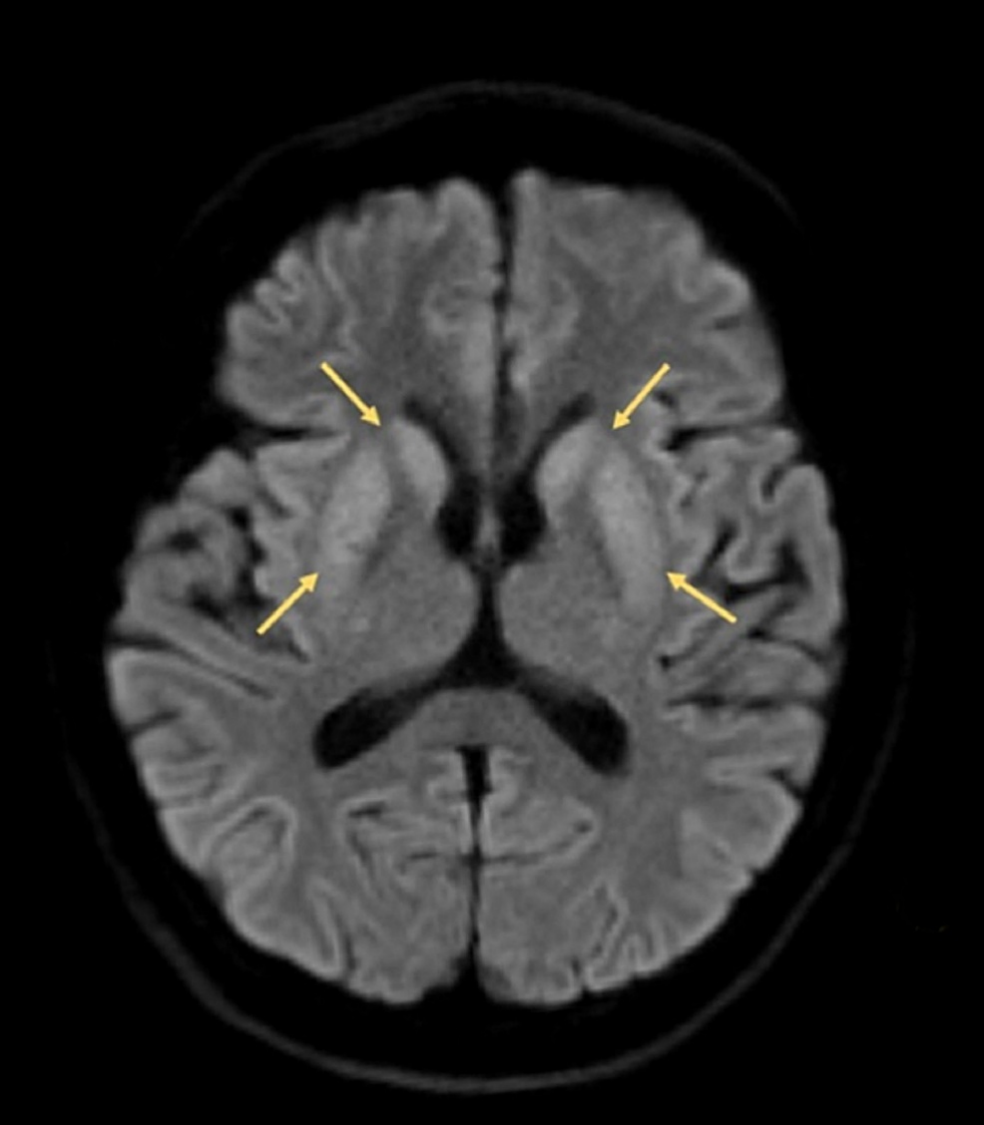
Brain MRI (DWI) on day two of hospital admission. The axial view of the DWI shows symmetrical high-intensity lesions at the bilateral head of caudate nucleus and putamen. DWI: diffusion-weighted imaging

During the stay in the general ward, sodium supplementation with 3% hypertonic saline and water restriction were given for treatment of severe hyponatremia. The patient’s serum sodium level was closely monitored. On day two after admission, the patient showed acute onset of altered mental status with agitation, limb rigidity, bradykinesia, and dystonia. Drug-related extrapyramidal side effects were first considered. As a result, the likely causative pharmaceutical agents (chlorpromazine, metoclopramide) were stopped. However, the patient’s condition did not improve. As a result, brain MRI was arranged. The conventional MRI sequence - T2 weighted image (T2WI), Fluid-attenuated inversion recovery (FLAIR) - showed unremarkable findings (Figure 1); however, diffusion-weighted imaging (DWI) revealed diffusion restriction lesions at the bilateral caudate nucleus and putamen (Figure 2).

**Figure 3:**
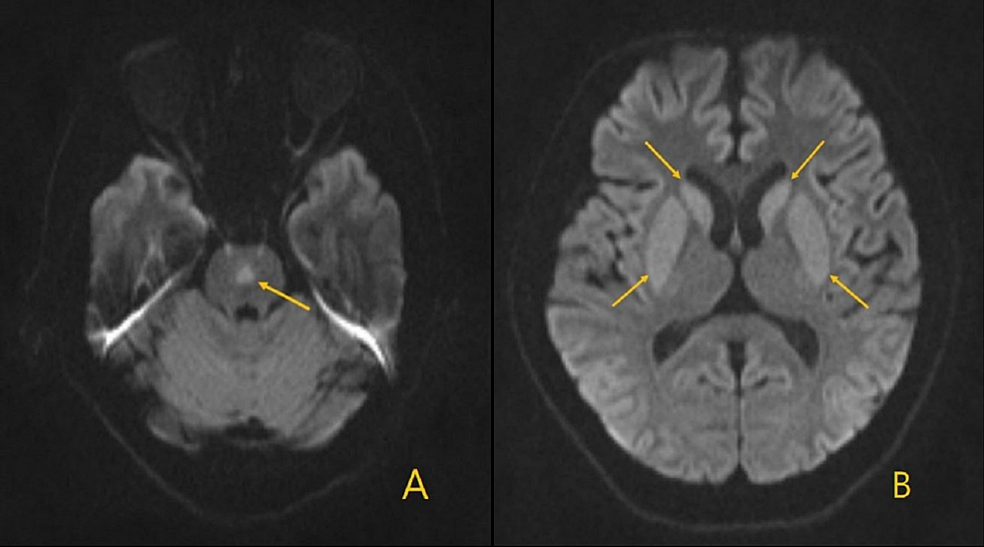
Brain MRI (DWI) on day 12 of hospital admission. The axial view of the DWI shows: (A) new high intensity lesion at the central pons, consistent with CPM; and (B) the previous lesions at the bilateral caudate nucleus and putamen did not subside and became more conspicuous. DWI: diffusion-weighted imaging; CPM: central pontine myelinolysis

EPM was highly suspected due to serum sodium correction. Subsequently, the correction rate for hyponatremia was adjusted. Treatment with Madopar, rasagiline, and a low-dose dopamine agonist (Mirapex) was given and carefully adjusted to treat the Parkinsonian features including bradykinesia, dysarthria, and rigidity. A follow-up brain MRI was performed on day 12 of the hospital stay and revealed a new diffusion restriction lesion at the central pons, which is the typical imaging feature of CPM. The previously identified lesions at the caudate nucleus and putamen did not subside and became more conspicuous in the second MRI (Figure 3). Overall, the patient’s general condition and Parkinsonism gradually improved over the course of treatment. The patient was discharged after one month of treatment and continued to receive rehabilitation at the outpatient department.

## Discussion

In the present case, the first diagnostic challenge was the osmotic demyelination syndrome at symptom onset. Although the patient was initially admitted for correction of severe hyponatremia, the unusual presentation with Parkinsonian movement disorder resulted in concerns about extrapyramidal symptoms, which are common drug side effects during hospitalization. The anatomical structures involved in EPM explain the Parkinsonian movement disorder [1,6]. This case suggests that EPM should be included in the differential diagnosis for acute parkinsonism or extrapyramidal symptoms - especially when the patient is being treated for hyponatremia.

In the course of osmotic demyelination syndrome, imaging findings are often significantly delayed after the clinical presentation. Compared with conventional CT, brain MRI is more sensitive for lesion detection [7]. Osmotic demyelination syndrome usually manifests as a low T1-weighted image (T1WI) signal intensity and high T2WI signal intensity in the affected area. Furthermore, there is no enhancement of lesions after intravenous gadolinium administration. In cases of CPM, an oval-shaped or trident-shaped high signal intensity abnormality in central pons is observed using T2-weighted or FLAIR sequences. In EPM, symmetrical high signal intensity abnormalities are seen in the bilateral caudate nucleus and bilateral putamen with sparing of the globus pallidus in T2-weighted and FLAIR sequences [7]. However, detection of lesions using conventional MRI sequences (T1WI, T2WI, FLAIR) usually takes 10-14 days after the onset of clinical symptoms and signs. In contrast, DWI can detect abnormal diffusion restriction in demyelinated lesions within 24 hours after initial onset, which can increase diagnostic confidence [7].

In the present case, the temporal changes on brain MRI from EPM to CPM are an important observation. Notably, previous studies have reported this condition [8,9]. According to the previously published cases, when EPM and CPM coexist, it seems that EPM precedes the event of CPM. The Parkinsonian movement disorder caused by EPM can be managed with dopaminergic treatment [1]; however, CPM can become a life-threatening condition when respiration is impaired. If the temporal changes in DWI findings with syndrome progression can be clarified, this information could be used to support early diagnosis and treatment. 

## Conclusions

Osmotic demyelination syndrome is a complication of rapid correction of severe hyponatremia. EPM is a subtype of osmotic demyelination syndrome that usually presents as Parkinsonian features, thus resulting in a diagnostic challenge. Further studies are needed to confirm the temporal progression of DWI changes from EPM to CPM in order to support early diagnosis and treatment.
